# Toxic accumulation of copper and neuropsychiatric symptoms due to a familial tunisian compound heterozygous ATP7B missense mutation

**DOI:** 10.1192/j.eurpsy.2021.1901

**Published:** 2021-08-13

**Authors:** N. Abdelmoula

**Affiliations:** Genomics Of Signalopathies At The Service Of Medicine, Medical University of Sfax, Sfax, Tunisia

**Keywords:** ATP7B, Copper, Neuro-psychiatric symptoms, Wilson disease

## Abstract

**Introduction:**

Wilson disease (WD) is an autosomal recessive genetic disorder caused by loss-of-function mutations in the P-type copper ATPase, ATP7B (ATPase copper-transporting beta), which transports copper out of cells. It is characterized by toxic accumulation of copper primarily in the liver and brain, leading to liver disorders and/or neuropsychiatric symptoms.

**Objectives:**

Here, we report a Tunisian pedigree associated to familial ATP7B gene mutation.

**Methods:**

Medical genetic investigations, and molecular screening of ATP7B gene mutations were performed to a Tunisian three-generation pedigree with eight members having neuropsychiatric symptoms. Molecular genetic testing of the ATP7B 21 exons was carried out by direct sequencing.

**Results:**

A compound heterozygote mutational status of ATP7B with 2 substitutions: p.H1069Q and p.D642H was found. The family originated from the city of Sfax (Tunisia) showed a pronounced amount of consanguinity and eight members affected by WD. All cases derived from consanguineous couples and harbored psychiatric disorders associated or not to neurologic symptoms. Diagnosis of WD was piloted first through the cases harbouring intention tremor in the upper limbs and ataxia associated with psychiatric symptoms.

**Conclusions:**

The first missense mutation p.H1069Q - c.3207C>A (CAC-CAA) (exon 14) is the most commonest mutation in WD associated with late onset neurological conditions in Europe (Natural variantiVAR_000758 dbSNP:rs76151636). The second missense mutation in exon 6 : p.D642H - c.1924G>C (GAC-CAC) (Natural variantiVAR_000713 dbSNP:rs72552285) affects the domain affinity to copper or the folding structure in the cytoplasmic region and decreases the stability, leading to abnormal localization of the protein within cytoplasm and an impairment of protein function.

**Disclosure:**

No significant relationships.

